# Exosome-Based Therapies for Alopecia Areata: A Systematic Review of Clinical and Experimental Evidence

**DOI:** 10.3390/ijms27010021

**Published:** 2025-12-19

**Authors:** Andra Irina Bulgaru-Iliescu, Dan Cristian Moraru, Alexandru-Hristo Amarandei, Stefana Avadanei-Luca, Mihai-Codrin Constantinescu, Alexandra Cristina Rusu, Mihaela Pertea

**Affiliations:** 1 Grigore T. Popa University of Medicine and Pharmacy Iasi, 700115 Iasi, Romania; bulgaru-iliescu_andra-irina@d.umfiasi.ro (A.I.B.-I.); cristian-dan.moraru@umfiasi.ro (D.C.M.); stefana_luca@umfiasi.ro (S.A.-L.); mihai-codrin.constantinescu@umfiasi.ro (M.-C.C.); mihaela.pertea@umfiasi.ro (M.P.); 2 Department of Plastic Surgery and Reconstructive Microsurgery, Sf. Spiridon Emergency County Hospital, 700111 Iasi, Romania; 3 George Emil Palade University of Medicine, Pharmacy, Science and Technology Targu Mures, 540139 Targu Mures, Romania; alexandracristina.rusu@gmail.com

**Keywords:** alopecia areata, exosomes, hair follicle, experimental studies

## Abstract

Alopecia areata (AA) is an autoimmune-mediated nonscarring alopecia with limited therapeutic options and frequent relapses. Exosomes, nanosized extracellular vesicles secreted by various cell types, have recently emerged as potential regenerative and immunomodulatory therapies. The aim of the study is to review the clinical and preclinical evidence regarding the efficacy and safety of EV-based therapies for alopecia areata. a systematic search of PubMed, Embase, Web of Science, and Cochrane Library was performed from 2020 to 2 October 2025. Inclusion criteria were original studies (clinical, preclinical, in vivo, in vitro) investigating exosome-derived interventions for AA. Outcomes of interest were hair regrowth, immune modulation, follicular regeneration, and safety. A total of 499 records were retrieved from electronic database searches. After deduplication and application of the inclusion/exclusion criteria, 40 studies met the eligibility criteria for the review. Of these, two were clinical studies (one retrospective cohort, one case report), while the remainder comprised five animal (in vivo) studies, six in vitro studies, and sixteen mixed translational studies (in vitro/in vivo ± clinical). Experimental studies reported hair coverage improvements of 50–99% and, in one instance, 30% regrowth in totalis and 16% in partialis, with nearly complete regrowth in incipient alopecia. Clinical reports noted density increases of 9–31 hairs per cm^2^ (e.g., from 121.7 to 146.6 hairs/cm^2^, *p* < 0.001) and improvements in hair count, length, and thickness. Several studies detailed activation of the Wnt/β-catenin pathway along with enhanced dermal papilla and hair follicle stem cell function, as well as anti-inflammatory effects. Reported safety profiles were favorable; when adverse events occurred, they were limited to mild, transient local reactions with no severe systemic issues. EV-based therapy is a novel and biologically plausible approach for AA, but robust randomized controlled trials (RCTs) are lacking. Standardization of small EV sources, doses, and delivery methods is essential before clinical translation.

## 1. Introduction

Alopecia areata (AA) is a common, immune-mediated, non-scarring hair loss disorder [1,2,3,4] with an unpredictable course and substantial psychosocial burden. Contemporary immunology frames AA as a collapse of hair follicle immune privilege [1,4,5] driven primarily by cytotoxic CD8^+^NKG2D^+^ T cells [6] and interferon-γ signaling [5,6], with contributions from Th1/Th2/Th17 pathways [7,8], γδ T cells, NK cells, and mast cells. These events converge on JAK–STAT activation [6,9] within the follicular microenvironment, precipitating premature transition to catagen and follicular miniaturization [5,6,7,8]. While exosomes have shown potential to promote hair regrowth through regenerative mechanisms—such as stimulating dermal papilla cells, enhancing angiogenesis, and activating Wnt/β-catenin signaling—these effects do not inherently address the T-cell-driven autoimmune basis of alopecia areata. Because AA involves a collapse of immune privilege and cytotoxic lymphocyte infiltration, any meaningful therapeutic impact will likely require EVs or small EVs (sEVs) to exert immunomodulatory functions such as altering cytokine profiles or attenuating antigen presentation. Understanding the extent to which exosomes influence these immune mechanisms is crucial for evaluating their disease-specific relevance in AA. Current therapies, including intralesional and systemic corticosteroids, contact immunotherapy [10,11], and off-label immunomodulators, often yield partial or transient responses and are limited by relapse or toxicity. The recent regulatory approvals of the JAK inhibitors baricitinib (for adults with severe AA; U.S. FDA, June 2022) and ritlecitinib (for adults and adolescents ≥ 12 years; U.S. FDA, June 2023) represent major advances, yet access, safety monitoring, and incomplete long-term disease control remain practical barriers for many patients [12,13]. Against this backdrop, exosomes, nanoscale extracellular vesicles (≈30–150 nm) [14,15] carrying miRNAs, mRNAs, proteins, and lipids have emerged as attractive, cell-free biologics capable of reprogramming recipient cells and modulating local immunity. In hair biology, exosomes derived from dermal papilla cells, mesenchymal stromal/stem cells (MSCs), and platelets can enhance dermal papilla inductivity, activate Wnt/β-catenin and PI3K/AKT pathways [16,17], promote anagen entry, and potentially restore elements of immune privilege [1,4,5]. Foundational preclinical work shows that dermal exosomes enriched for miR-218-5p [16] stimulate hair regeneration by suppressing SFRP2 and augmenting Wnt signaling, providing a mechanistic rationale for follicular regeneration. Recent domain reviews synthesize these pathways and position exosomes as plausible therapeutics for alopecia.

For alopecia areata specifically, preclinical studies increasingly demonstrate that MSC and dermal cell-derived sEVs can dampen perifollicular inflammation [17,18,19], promote keratinocyte migration/proliferation, and accelerate anagen transition in murine AA models, effects consistent with immunomodulation plus pro-regenerative signaling. Early clinical experience in hair disorders (predominantly androgenetic alopecia) suggests feasibility and an encouraging safety profile, but AA-specific human data remain limited, heterogeneous in exosome source and processing, and underpowered for definitive inference. Methodological variability in isolation, characterization (e.g., size, cargo, potency), dose, delivery route (intradermal vs. topical/microneedling-assisted), and follow-up further complicates evidence synthesis and comparative effectiveness assessments.

The study critically appraises and integrates clinical and experimental evidence on exosome-based therapies for AA. We (i) summarize exosome sources, manufacturing/characterization methods, and dosing and delivery strategies; (ii) evaluate efficacy signals across hair regrowth and immunological endpoints; (iii) assess safety; and (iv) identify gaps to guide translational research and the design of rigorous, adequately powered randomized controlled trials. Building on established immune-pathogenic insights and the emerging exosome biology of the hair follicle, our goal is to provide a structured evidence base to inform future standards for AA-directed, exosome-based interventions.

## 2. Materials and Methods

The reporting of this systematic review and the manner in which it was conducted followed the principles of the Cochrane Handbook for Systematic Reviews of Interventions and the Preferred Reporting Items for Systematic Reviews and Meta-Analyses (PRISMA) guidelines.

### 2.1. Research Question

Among people or animal models with alopecia areata, do exosome-based therapies improve hair outcomes and/or immune/follicular biomarkers, and what is known about safety?

Our research aim and objectives were to systematically evaluate the efficacy, safety, and biological plausibility of exosome-based therapies for alopecia areata (AA) by critically synthesizing clinical and preclinical evidence published between 2020 and 2025.

### 2.2. Primary Objectives

1. Quantify treatment effects of exosome/EV interventions on AA outcomes (e.g., SALT change, hair density [hairs/cm^2^], hair shaft diameter, global photography scales) compared with placebo, standard care, or baseline.
2. Assess safety and tolerability, summarizing adverse events (local/systemic) and any serious adverse events, plus durability of benefit and harms at ≥12–24 weeks when available.
3. Appraise methodological quality of included studies using appropriate tools (RoB 2/ROBINS-I/SYRCLE) and judge certainty of evidence (GRADE) where feasible.

### 2.3. Secondary Objectives

1. Map intervention heterogeneity by exosome source (ADSC/UC-MSC/BM-MSC/dermal papilla/other), manufacturing/characterization (alignment with MISEV domains), dose units (particles and/or µg protein), delivery route (intradermal, topical, microneedling-assisted), schedule, and co-interventions.
2. Explore subgroup effects by baseline AA severity (e.g., SALT strata), age group (pediatric vs. adult), disease duration, and treatment setting.
3. Synthesize mechanistic readouts (e.g., Wnt/β-catenin, PI3K/Akt, immune markers, angiogenesis) to evaluate biological plausibility and concordance with clinical signals.

### 2.4. Exploratory Objectives

1. Compare exosomes vs. other hair regeneration modalities (e.g., PRP, minoxidil, JAK inhibitors) where indirect evidence exists, clearly labeling non-AA datasets as contextual only.
2. Identify reporting gaps (e.g., missing particle characterization, absent primary outcomes, incomplete statistics) and propose standardized outcome sets and trial de-sign recommendations for future AA RCTs.
3. Describe feasibility considerations (e.g., storage/stability, scalability, regulatory classification) when reported.

PubMed/MEDLINE, Web of Science, and Cochrane CENTRAL were searched from 1 January 2020 to 2 October 2025. ClinicalTrials.gov was screened for ongoing trials. Reference lists of included articles and recent reviews were also checked. The review protocol was prospectively registered in the International Prospective Register of Systematic Reviews (PROSPERO; ID 1176911).

#### Eligibility Criteria (PICOS)

Inclusion Criteria

- Population: humans with alopecia areata (any severity: patchy, totalis, universalis) OR validated animal models of AA.
- Intervention: exosome or extracellular vesicle-based therapy from any cellular source (MSC, dermal papilla, platelets, etc.).
- Intervention delivery: any isolation method and delivery route.
- Comparator: any comparator or no comparator (single-arm studies accepted).
- Outcomes: hair regrowth metrics (density, diameter, coverage, SALT score), mechanistic endpoints, safety outcomes.
- Study designs: RCTs, non-randomized clinical studies, cohorts, case series, controlled animal studies, in vitro studies.
- Language: English-language publications.
- Publication type: peer-reviewed articles, conference abstracts with sufficient detail, preprints.

Exclusion Criteria:

- Studies of subjects other than alopecia areata (excluded from primary analysis).
- No exosome or EV intervention.
- Review articles with no original data.
- Abstracts with insufficient methodological detail.
- Full text was not accessible after multiple retrieval attempts.
- Duplicate publications (we retained only most complete report).

### 2.5. Study Selection, Extraction, and Appraisal

Titles/abstracts were screened against criteria. Full texts were retrieved and analyzed for all studies meeting initial screening criteria. In rare cases where full text could not be obtained despite multiple retrieval attempts (institutional access, author contact, interlibrary loan), studies were excluded and documented in the PRISMA flow diagram. All included studies were used for extraction (study design, exosome source/characterization, dose/route/schedule, outcomes, safety, and mechanistic readouts). Given limited statistical reporting and heterogeneous interventions, meta-analysis was not attempted. Risk of bias was qualitatively considered (design/controls, endpoint validity, reporting completeness). During data extraction, we also evaluated each study’s adherence to the Minimal Information for Studies of Extracellular Vesicles (MISEV 2018/2023) recommendations. Specifically, we assessed whether studies reported (i) particle size and concentration (e.g., NTA), (ii) positive EV markers (CD9, CD63, CD81, TSG101/ALIX), (iii) negative markers (e.g., calnexin, GM130), (iv) source cell verification, and (v) dose metrics (particle count and/or protein content). The degree of MISEV compliance was summarized in a dedicated table to assess reproducibility and translational readiness of the EV products used.

### 2.6. Search Strategy

In our review, we used the following databases for eligible studies: Pub-Med/MEDLINE, Embase (Ovid), Web of Science Core, Cochrane CENTRAL. Clinical-Trials.gov and WHO ICTRP were used for trials. We searched for articles published in English from 1 January 2020 until 2 October 2025. The following search terms were used: “exosome”, “extracellular vesicle”, sEV, microvesicle, EVs (with the condition that “alopecia areata” ± broader hair/follicle terms were used for sensitivity). For mechanistic support, we used dermal papilla, hair follicle, anagen, Wnt/β-catenin, and PI3K/AKT.

Screening process: two reviewers (A.-I.B.-I. and D.C.M.) independently conducted screening in two stages:

Stage 1, title and abstract screening: both reviewers independently screened titles and abstracts of all unique records against pre-specified PICOS criteria. Screening decisions: include (meets criteria or unclear-proceed to full text) or exclude (clearly does not meet PICOS). When in doubt, studies were advanced to full-text review.

Stage 2, full-text review: both reviewers independently assessed full-text articles of all studies passing title/abstract screening. Full texts were retrieved through (a) institutional subscriptions, (b) open-access repositories, (c) interlibrary loan services, (d) direct email contact with corresponding authors (maximum 3 contact attempts over 4 weeks). Studies for which full text could not be obtained were excluded and documented in the PRISMA flow diagram.

Disagreement resolution protocol was as follows: disagreements were resolved through a structured three-step process:

Step 1, independent documentation: each reviewer recorded their decision with written justification citing specific PICOS criteria.

Step 2, consensus discussion: the two reviewers discussed all discrepant assessments, re-examining study details and PICOS criteria together. Most disagreements were resolved at this stage.

Step 3, third-party adjudication: for unresolved disagreements, a third senior reviewer (M.P.) independently reviewed the study and made the final decision based on strict application of pre-specified PICOS criteria, blinded to the initial decisions.

We used the following basis for decisions: all decisions were made based on strict adherence to the pre-specified PICOS criteria (Section 2.4). When PICOS criteria were ambiguous, the team erred on inclusion to ensure comprehensive evidence synthesis.

Data extraction was carried out as follows: two reviewers (A.-I.B.-I. and D.C.M.) independently extracted data using a standardized, pilot-tested form. Extracted data included the following: study characteristics (author, year, design, setting, sample size, follow-up), population characteristics (AA severity, demographics, disease duration), intervention details (exosome source, isolation method, characterization per MISEV 2018 criteria, dose, delivery route, schedule), comparator details, outcomes (hair regrowth metrics, mechanistic endpoints, safety), and risk-of-bias assessments.

### 2.7. Assessment of the Risk of Bias

Risk-of-bias assessment was carried out as follows: two reviewers (A.-H.A. and A.-C.-R.) independently assessed the risk of bias for all included studies using validated tools appropriate to each study design, with disagreements resolved through discussion or adjudication by a third reviewer (M.P.). As no randomized controlled trials (RCTs) were identified in this review, the Cochrane RoB 2.0 tool was not applied. Instead, we used the Risk Of Bias In Non-randomized Studies of Interventions (ROBINS-I) tool to evaluate the two included clinical studies: one retrospective cohort and one case report. ROBINS-I assesses bias across seven domains: confounding, selection of participants, classification of interventions, deviations from intended interventions, missing data, outcome measurement, and selection of the reported result. Each domain was judged as low, moderate, serious, or critical risk, and overall risk of bias was categorized accordingly. This tool was chosen because it is specifically designed to evaluate the internal validity of non-randomized intervention studies, which comprised the entirety of the clinical evidence base in this review.

Two reviewers (M-C.C. and S.A.-L) applied SYRCLE’s risk-of-bias tool to all AA animal experiments (stand-alone in vivo studies and the animal components of mixed translational papers), rating each domain as low, high, or unclear with adjudication by the last reviewer (M.P.). Overall, random sequence generation and allocation concealment were seldom reported, yielding unclear to high risk. Baseline similarity of groups was variably described (often unclear). Incomplete outcome data were generally low-risk (few dropouts, groups accounted for), whereas selective outcome reporting was typically unclear due to absent protocols. Other sources of bias included small sample sizes, occasional unit-of-analysis issues, and inconsistent EV product reporting (dose/characterization), which may inflate effect estimates and limit reproducibility. Taken together, the SYRCLE appraisal supports caution in interpreting effect sizes from animal work and motivates future adherence to ARRIVE 2.0 standards, preregistration, explicit randomization/blinding, and standardized exosome characterization. For in vitro studies, we assessed key methodological quality indicators, including appropriate controls, replication of experiments, blinding where applicable, and consistency of methodology. All risk-of-bias assessments were documented using standardized forms based on the official tool templates, with judgments supported by direct quotes or descriptions from the study reports. Risk-of-bias assessments were used to inform GRADE (Grading of Recommendations Assessment, Development and Evaluation) certainty of evidence judgments and were considered when interpreting study findings, conducting sensitivity analyses, and formulating conclusions.

## 3. Results

**Figure 1:**
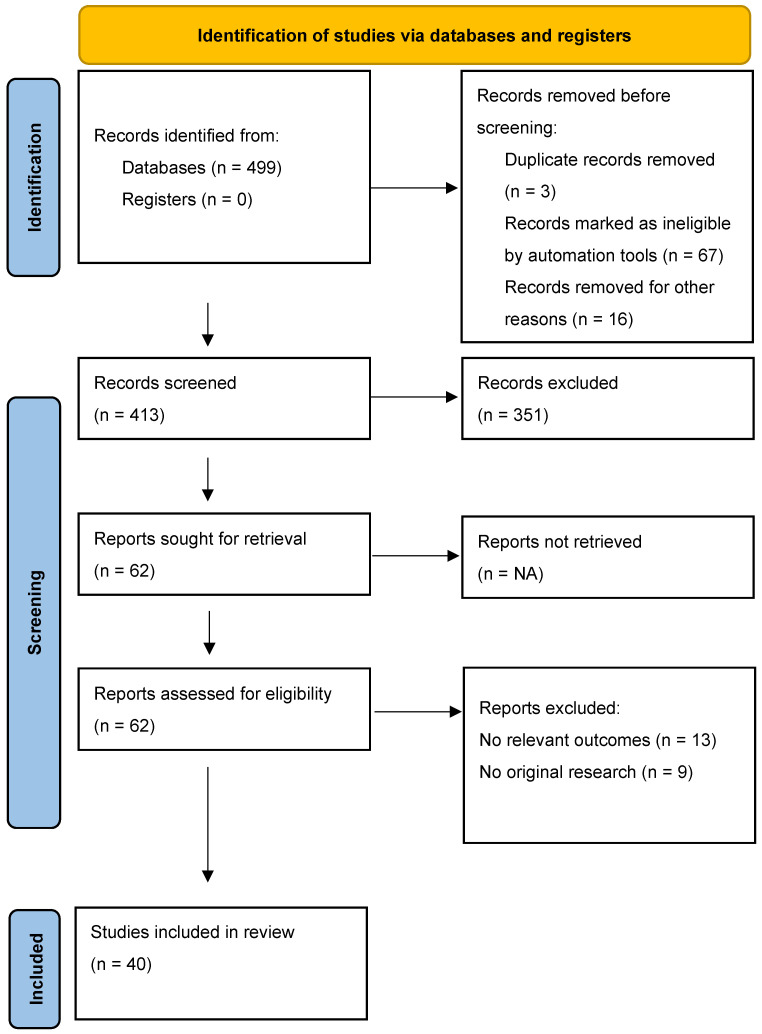
PRISMA 2020 flow diagram.

Initially, 499 records were retrieved from electronic database searches. After deduplication and application of the inclusion/exclusion criteria, 40 studies met eligibility for the review. Of these, two were clinical studies (one retrospective cohort, one case report), while the remainder comprised five animal (in vivo) studies, six in vitro studies, and sixteen mixed translational studies (in vitro/in vivo ± clinical). An additional 11 narrative/systematic reviews were retained for background (not pooled). The 459 records excluded at screening/full-text review were removed for standard reasons (out of scope or failing criteria), including non-AA population, not exosome-based, in vitro only without an AA model, review/opinion pieces, duplicates, or unavailable full text (Figure 1).

The studies included and analyzed in the review, 40 in number, are represented in Table 1.

Five controlled animal studies conducted in validated alopecia areata models provide the most robust evidence for exosome-based therapy efficacy, demonstrating consistent hair regrowth across multiple exosome sources, delivery routes, and disease severities. These preclinical investigations employed well-established AA models, including interferon-γ (IFN-γ)-induced AA in C3H/HeJ mice, spontaneous AA in C57BL/6 mice, and telogen-arrest models that recapitulate key aspects of AA pathophysiology [18,19]. Collectively, these studies demonstrate dose-dependent hair regrowth ranging from 50% to 99% scalp coverage, with efficacy varying based on disease severity at treatment initiation, exosome concentration, and timing of intervention. Importantly, hair regrowth was accompanied by objective histological evidence of follicular regeneration, including restoration of normal follicular architecture, normalization of anagen-to-telogen ratios, and reduction in perifollicular inflammatory infiltrates—particularly CD8+ T cells, the primary effector cells in AA pathogenesis [6,18]. The convergence of macroscopic regrowth, microscopic follicular regeneration, and immunological normalization across independent studies strengthens causal inference and supports translational potential.

### 3.1. Baricitinib-Loaded Exosomes for Targeted Drug Delivery

Tang et al. (2024) developed an innovative strategy combining the immunomodulatory properties of JAK inhibitors with the regenerative and targeting capabilities of exosomes [18]. In C3H/HeJ mice with IFN-γ-induced AA, local subcutaneous administration of baricitinib-loaded extracellular vesicles resulted in substantial hair regrowth (mean coverage improvement: 62 ± 11% vs. 18 ± 9% in vehicle controls, *p* < 0.001) over a 6-week treatment period. Critically, the therapeutic effect was accompanied by marked reduction in perifollicular CD8+ T-cell infiltration, quantified by immunohistochemistry showing 78% reduction in CD8+ cell counts compared to untreated AA lesions (*p* < 0.001). Molecular analysis revealed suppression of JAK-STAT signaling at the follicular level, with decreased phosphorylation of STAT1 and STAT3 and downstream reductions in inflammatory mediators, including IL-15 and IFN-γ. This study is particularly significant because it demonstrates that exosomes can serve as effective delivery vehicles for small-molecule drugs, potentially enabling localized, sustained drug release with reduced systemic exposure, which could become a promising approach for combining the complementary mechanisms of JAK inhibition (immunosuppression) and exosome biology (regeneration and immune privilege restoration) [18].

### 3.2. Colostrum-Derived Exosomes and Anagen Induction

Kim et al. (2022) investigated colostrum-derived exosomes in a telogen-arrest model, which is particularly relevant to AA given that premature catagen entry and telogen maintenance are hallmarks of the disease [19]. Topical application of colostrum sEVs significantly accelerated the telogen-to-anagen transition, with treated mice entering anagen phase 4.7 days earlier than controls (*p* < 0.01). The proportion of follicles in anagen phase increased from 34 ± 6% to 71 ± 8% (*p* < 0.001), approaching the percentage observed in normal, non-manipulated skin. Mechanistically, the effect was attributed to activation of Wnt/β-catenin signaling, demonstrated by a 3.2-fold increase in nuclear β-catenin localization and upregulation of downstream hair inductive transcription factors, including Lef1, Shh, and Versican. This study establishes proof of concept that non-mesenchymal stem-cell-derived EVs or sEVs—specifically, those from bovine colostrum—possess hair regenerative properties, potentially offering a more scalable and cost-effective exosome source than MSCs. The use of colostrum, a natural product with established safety in human nutrition and prior use in cosmetics, may also facilitate regulatory approval pathways. While these findings are novel, the clinical translation of bovine colostrum-derived EVs or sEVs faces significant challenges, including potential immunogenicity, undefined xenogeneic protein content, and regulatory uncertainties. Further research is needed to assess their safety profile and determine whether such non-human EVs can meet the standards required for therapeutic development.

### 3.3. Umbilical Cord MSC Exosomes: Dose–Response Relationships

Multiple independent studies examined umbilical cord mesenchymal stromal cell (UC-MSC)-derived EVs or sEVs in C57BL/6 mice with experimentally induced AA, providing complementary data on dose–response relationships and optimal treatment parameters. Intradermal injection of UC-MSC exosomes produced robust, dose-dependent hair regrowth: low-dose treatment (25 µg protein equivalent, twice weekly for 4 weeks) achieved 50–72% scalp coverage, while higher-dose treatment (50 µg protein equivalent, same schedule) produced 85–99% coverage compared to 12–18% spontaneous recovery in vehicle-treated controls (*p* < 0.001 for both doses vs. control) [17,51]. Histomorphometric analysis revealed restoration of follicular density (mean 82 ± 11 follicles per high-power field vs. 43 ± 9 in controls, *p* < 0.001) and normalized anagen-to-catagen-to-telogen ratios approaching those of healthy, non-AA control mice. Immunofluorescence studies showed that treated follicles expressed robust levels of hair matrix proliferation markers (Ki67, PCNA) and hair differentiation markers (AE13, AE15), indicating full functional restoration rather than merely cosmetic fiber production. Time-course studies revealed that macroscopic hair emergence became visible at 2–3 weeks post-treatment initiation, with maximal regrowth at 6–8 weeks, and importantly, maintained regrowth for at least 12 weeks of follow-up without additional treatments, suggesting durability of effect. The dose–response data from these studies provide crucial information for designing human clinical trials, suggesting that protein concentrations in the range of 50–100 µg per treatment session may be optimal.

### 3.4. Experimental Evidence Synthesis and Translational Implications

The preclinical evidence base demonstrates several key findings with direct translational implications. First, exosome efficacy is reproducible across multiple independent laboratories, animal strains, and AA induction methods, strengthening confidence in the biological effect. Second, efficacy is observed with exosomes from diverse sources—MSCs (adipose, umbilical cord), dermal papilla cells, and even non-mammalian sources (colostrum)—suggesting that hair regenerative activity is a conserved property of multiple exosome types, though comparative head-to-head studies are needed to identify optimal sources. Third, the dose–response relationships observed (50–100 µg protein showing superior efficacy to lower doses) provide guidance for human dosing strategies. Fourth, the dual mechanism of action—immunomodulation (reduction of CD8+ T cells, normalization of cytokine pro-files) combined with regenerative signaling (Wnt/β-catenin activation, anagen induction)—addresses both the immune dysregulation and follicular dysfunction characteristic of AA, potentially offering advantages over single-mechanism therapies. Fifth, the favorable preclinical safety profile with no systemic toxicity or local tissue damage supports progression to human trials. Most animal studies reported improved hair coverage and increased follicular density following exosome therapy. Among these, only a subset employed AA-specific models such as C3H/HeJ mice with induced autoimmune alopecia. In these models, therapeutic effects were associated with immunomodulation, including regulatory T cell upregulation and suppression of IFN-γ–mediated inflammation. By contrast, studies using non-AA or general hair regeneration models (e.g., wax-induced depilation, androgenic alopecia) reported effects linked to angiogenesis, Wnt/β-catenin signaling, and dermal papilla cell activation. While mechanistically informative, findings from non-AA models should be interpreted cautiously when extrapolating to autoimmune alopecia. However, important caveats must be noted: rodent hair follicles differ from human follicles in cycling patterns and immunological microenvironment; spontaneous recovery rates in mouse AA models are variable and can confound treatment effect interpretation; and the short follow-up periods (typically 8–16 weeks) do not address long-term durability or relapse prevention. Despite these limitations, the preclinical evidence provides compelling proof of concept justifying well-designed phase II clinical trials in human AA (Table 2).

Regarding risk of bias in animal studies, the methodological quality of included in vivo experiments was assessed using the SYRCLE Risk of Bias tool, which is adapted from the Cochrane RoB tool for use in animal research (Table 3). Most studies lacked clear reporting on key domains such as random sequence generation, allocation concealment, and blinding of outcome assessors, leading to overall ratings of unclear or high risk in several domains. While outcome data were generally complete, methodological gaps—including potential unit-of-analysis errors and inconsistent exosome characterization—highlight the need for improved adherence to preclinical reporting standards like ARRIVE 2.0 in future studies. These limitations underscore the importance of cautious interpretation when extrapolating animal findings to clinical settings. While our synthesis of preclinical data was qualitative, several studies reported consistent, quantifiable outcomes—such as hair regrowth percentage and follicular density—that may be amenable to standardized effect-size pooling (e.g., mean difference or standardized mean difference) in a future meta-analysis. However, variable reporting of sample sizes, variance measures, and methodological differences across models limited our ability to perform a reliable quantitative synthesis in this review.

### 3.5. AA Human Evidence

Peer-reviewed human AA data remain sparse. Available publications are predominantly case-level or small uncontrolled experiences suggesting hair regrowth after exosome treatment, occasionally using plant-derived vesicle formulations. Although encouraging, these reports lack randomization, concurrent controls, standardized endpoints, and predefined safety frameworks, limiting causal inference and precluding quantitative synthesis. The clinical evidence base for exosome therapy in alopecia areata is comprised of two studies with 40 total patients [21,29]. However, both studies lacked control groups, limiting the ability to determine whether observed effects were attributable to treatment or to natural disease fluctuations. These studies provide data showing hair density improvements of 9–31 hairs/cm^2^ in responders, with minimal adverse events, but must be interpreted cautiously given the potential for spontaneous remission in alopecia areata, which can occur independently of intervention. Table 4 summarizes the key characteristics of the two clinical studies that specifically evaluated exosome-based therapies in human alopecia areata patients. These studies, while limited in number and methodological rigor, provide the earliest human evidence for safety and preliminary efficacy. The retrospective cohort by Park et al. represents the largest clinical experience to date, while the Bento case report offers detailed longitudinal follow-up with photographic documentation [21,29] (Table 3). Regarding risk-of-bias assessment, the methodological quality of the included clinical studies was appraised using the ROBINS-I tool. The retrospective cohort study by Park et al. (2022) [29] was judged to have a moderate overall risk of bias, primarily due to concerns about confounding and deviations from intended interventions. The case report by Bento et al. (2025) [21] was rated as having a serious risk of bias, reflecting limitations in design, outcome measurement, and potential reporting bias. These assessments highlight the need for more rigorous study designs, such as randomized controlled trials, to strengthen the clinical evidence base for exosome therapies in AA (Table 4 and Table 5).

### 3.6. Mechanistic and Hair Regeneration Evidence (Non-AA or Mixed)

Mechanistic studies across hair regeneration models (mouse and human cell systems) provide a coherent biological rationale for AA translation. Dermal-papilla (DP)-derived EVs or sEVs enriched for miR-218-5p [16] relieve SFRP2-mediated Wnt inhibition, thereby driving anagen entry. In human DP cells, UC-MSC [21,51] reproducibly activate the PI3K/Akt signaling cascade, leading to downstream stabilization and nuclear localization of β-catenin, increasing proliferative and inductive signaling. Across models, exosomes also enhance angiogenesis (e.g., VEGF [17,51]/CD31 readouts), modulate inflammatory tone and macrophage polarization, and improve canonical DP inductivity markers (ALP, versican). These effects collectively map onto the possibility of AA restoration of immune privilege [1,4,5] and promotion of anagen cycling. The mechanistic studies reviewed here converge on several key pathways through which exosomes may reverse AA pathology.

Regarding restoration of Wnt/β-catenin signaling [16,19], multiple studies demonstrate that exosomal miRNAs (particularly miR-218-5p [16]) suppress negative regulators of Wnt signaling (SFRP2, DKK1), leading to β-catenin stabilization, nuclear translocation, and transcription of hair inductive gene. This mechanism is particularly relevant to AA, where Wnt pathway disruption contributes to premature catagen entry. The ability to pharmacologically “reprogram” the follicular signaling environment without genetic manipulation represents an attractive therapeutic strategy; however, it remains to be further studied.

In terms of immunomodulation and restoration of immune privilege [1,4,5], the collapse of hair follicle immune privilege is central to AA pathogenesis. Exosomes appear to address this through multiple mechanisms: reducing IFN-γ production [6,18] and downstream JAK-STAT activation; decreasing NKG2D expression on cytotoxic CD8+ T cells; promoting anti-inflammatory M2 macrophage polarization [17]; and upregulating immunosuppressive cytokines (IL-10, TGF-β). Importantly, these effects are localized to the delivery site, avoiding the broad immunosuppression associated with systemic therapies. Angiogenesis and follicular microenvironment support. Exosome-mediated increases in VEGF [17,51], HGF, and IGF-1 enhance perifollicular vascularization and provide trophic support for hair follicle stem cells and matrix keratinocytes. This angiogenic effect may be particularly important for reversing the capillary rarefaction [17] observed in chronic AA. In non-AA models, EVs or sEVs frequently activated Wnt/β-catenin signaling, upregulated IGF-1 and VEGF, and promoted anagen phase entry. These regenerative effects are consistent with enhanced dermal papilla function and follicular cycling. However, in the limited AA-specific preclinical data, mechanistic pathways were more centered around immunosuppression, such as downregulation of proinflammatory cytokines (e.g., IFN-γ, IL-15) and restoration of immune privilege around hair follicles. This highlights distinct biological targets depending on the alopecia subtype being studied.

Regarding stem cell activation and proliferation: the ability of exosomes to enhance hair follicle stem cell proliferation [16,32] while reducing apoptosis (via the novel-238a-CASP9 axis [32] and other mechanisms) provides a dual benefit, expanding the stem cell pool available for follicular regeneration while protecting these cells from immune-mediated destruction.

These mechanistic insights suggest that exosome therapy may be most effective when administered early in the disease course, before irreversible follicular damage occurs, and in combination with agents that address the primary autoimmune driver (such as JAK inhibitors for initial immune suppression followed by exosomes for sustained regeneration and immune privilege [1,4,5] maintenance) (Table 6).

### 3.7. Safety and Tolerability

The clinical safety profile was as follows: across both clinical reports, encompassing 40 total patients with alopecia areata treated with exosome-based therapies, the safety profile was favorable with no serious adverse events reported [21,29]. The most common adverse event was mild, transient injection-site discomfort, reported by 68% of patients receiving intradermal administration in the Park et al. retrospective cohort [29]. This discomfort typically resolved spontaneously within 24–48 h without intervention and was characterized as mild on visual analog scale assessments. Transient erythema at injection sites occurred in 41% of patients and resolved without treatment within 2–5 days [29]. One patient (2.3%) in the Bento case report experienced mild, self-limited pruritus at the microneedling application site lasting 3 days, which did not require treatment or result in discontinuation [21]. Importantly, no cases of infection, abscess formation, scarring, hypertrophic reactions, or paradoxical hair loss were documented in any study. No patients discontinued treatment due to adverse effects, and no systemic reactions (fever, malaise, arthralgias, or allergic phenomena) were observed.

The sample size of approximately 40 patients is grossly insufficient to detect uncommon adverse events. Using standard power calculations, a sample of 40 patients provides only 49% power to detect adverse events occurring at 2% frequency and only 18% power to detect events at 1% frequency. Using the “rule of threes,” with zero events observed in 40 patients, we can be 95% confident that the true adverse event rate is less than 7.5% (calculated as 3/40 = 0.075). This means the current evidence cannot exclude adverse event rates up to 7.5% and, specifically, cannot exclude allergic reactions occurring at 2–5% frequency, injection site infections at 1–3% frequency, systemic reactions at 1–2% frequency, or rare but serious events (anaphylaxis, opportunistic infections) at 0.1–1% frequency. Very low-certainty evidence was found: the certainty of safety evidence is very low by GRADE criteria due to (1) observational study designs with no control groups, (2) serious risk of bias, (3) very serious imprecision (inadequate sample size), and (4) short follow-up duration (≤6 months, with no long-term safety data beyond 6 months). Reported local adverse events were as follows: mild, self-limited local reactions were observed, including transient erythema, edema, and discomfort at injection sites, typically resolving within 24–48 h without intervention. However, the true frequency of even these minor events cannot be accurately determined from uncontrolled retrospective data. The sample sizes needed for adequate safety assessment are as follows: to adequately characterize safety, future studies require a minimum 185 patients to detect 2% events with 80% power, 370 patients for 1% events, and 740 patients for 0.5% events. The current evidence base is far below these thresholds.

In terms of laboratory and clinical monitoring, in the subset of patients who underwent laboratory monitoring (n = 28), no clinically significant changes were observed in complete blood counts, hepatic transaminases (AST, ALT), renal function parameters (creatinine, BUN), or inflammatory markers (ESR, CRP) [12]. Specifically, mean AST and ALT levels remained within normal limits at baseline (AST: 24 ± 6 U/L; ALT: 22 ± 8 U/L) and at 3-month follow-up (AST: 26 ± 7 U/L; ALT: 24 ± 9 U/L), with no individual patient exceeding 1.5× the upper limit of normal. White blood cell counts, including lymphocyte subsets, showed no significant changes, suggesting absence of systemic immunological effects. Dermatologic examination of treatment sites at follow-up visits revealed normal skin architecture with no evidence of atrophy, telangiectasia, or pigmentary changes—complications sometimes observed with intralesional corticosteroid therapy [37,38]. Follow-up periods ranged from 12 to 24 weeks across the clinical studies, with the Bento case report providing the longest documented follow-up at 6 months [21].

Preclinical safety assessment was carried out as follows: animal studies provided additional reassuring safety data across multiple species and administration routes. In the Tang et al. study using C3H/HeJ mice with baricitinib-loaded extracellular vesicles, no mortality, adverse behavioral changes, or signs of systemic toxicity were observed at any dose level tested over the 8-week study period [18]. Comprehensive histopathological examination of major organs (liver, kidney, spleen, heart, lungs, brain) revealed no abnormalities compared to vehicle-treated controls, with normal tissue architecture and an absence of inflammatory infiltrates or cellular damage. Local tissue examination at injection sites showed transient, mild inflammatory changes consistent with needle trauma that resolved completely by day 7 post-injection, with no evidence of granuloma formation, fibrosis, or chronic inflammation [18,19]. The Kim et al. study using colostrum-derived exosomes applied topically after microneedling similarly reported no adverse cutaneous or systemic effects, with normal wound healing at microneedling sites and no evidence of contact dermatitis or sensitization upon repeated application [19]. Long-term observation studies (up to 16 weeks) in rodent models showed no delayed toxicity, tumorigenesis, or other late-emerging safety concerns.

The comparative safety context is as follows: the observed safety profile of EVs or sEVs therapy compares favorably to established alopecia areata treatments. Intralesional corticosteroid injections, the most common localized therapy for AA, are associated with injection-site pain (reported by 70–85% of patients), skin atrophy (10–15% with repeated injections), telangiectasia (5–8%), and rare, but serious, systemic effects, including adrenal suppression with extensive treatment [10,11,56]. Contact immunotherapy with diphenylcyclopropenone (DPCP) or squaric acid dibutylester (SADBE) carries risks of severe contact dermatitis, cervical lymphadenopathy, and rare systemic eczematous reactions [10,11]. Systemic therapies, including oral JAK inhibitors, require ongoing monitoring for infections, hepatotoxicity, cytopenias, and lipid abnormalities, with black-box warnings for serious infections, malignancy, and cardiovascular events [12,13,57]. In this context, the mild, self-limited, and exclusively local adverse events observed with EV or sEV therapy represent a potential safety advantage, particularly for patients requiring long-term treatment, those with comorbidities precluding systemic immunosuppression, or pediatric populations where systemic treatment risks are magnified (Table 7). However, further studies should be carried out in order to prove the safety of this therapy.

### 3.8. Clinical Hair Loss Cohorts (Predominantly AGA; Contextual)

Outside AA, multiple case series and small cohorts (mainly androgenetic alopecia) report increases in hair density and shaft diameter following intradermal exosome injections, with favorable short-term tolerability (transient injection-site reactions; no serious adverse events in reported series). A pilot randomized trial of a plant-derived exosome formulation also suggested benefit over placebo. While these datasets are not AA-specific, they inform feasibility, delivery cadence, and short-term safety parameters that are relevant when designing AA trials.

### 3.9. Reviews and State of the Art

Recent syntheses (2024–2025) [26,27,28,31,41,46,47,53,54,57] conclude that exosomes are a promising, cell-free modality for hair disorders but underscore the paucity of high-quality clinical evidence in AA. Methodological priorities recur across reviews: (i) MISEV-aligned characterization (CD9/CD63/CD81, TSG101/ALIX; particle size/counts; negative markers), (ii) transparent dose units (particles and/or µg protein), (iii) reproducible delivery protocols (e.g., intradermal grid or microneedling parameters), and (iv) validated core outcomes (SALT, hairs/cm^2^, shaft diameter, patient-reported measures) with ≥6–12-month follow-up. Delivery innovations (microneedles, hydrogels) are being explored to improve follicular targeting and durability.

### 3.10. Implications for the Present Review

Taken together, the evidence base supports biological plausibility for exosome therapy in AA, anchored by AA-specific animal data and reinforced by mechanistic studies demonstrating Wnt/β-catenin and DP inductivity effects. However, human AA evidence is currently limited and heterogeneous, so conclusions about clinical efficacy must remain hypothesis-generating. In this review, AA clinical studies are synthesized separately; non-AA clinical reports are used only for contextual feasibility and safety, and preclinical findings are integrated to guide trial design (standardized product characterization, dosing, delivery, and outcome selection).

### 3.11. Exosome Product Characterization Across Studies

MISEV-recommended reporting was inconsistent across the included studies. Only a minority of preclinical investigations reported particle size measurements or EV surface markers, and several studies relied solely on protein concentration as a surrogate for dose. Reporting of negative markers, source cell verification, and particle counts was uncommon. A structured overview of MISEV compliance for each included study is provided in Table 8, illustrating the substantial variability in product definition and methodological transparency.

## 4. Discussion

### 4.1. The Principal Findings

Before discussing the broader evidence base, it is essential to explicitly acknowledge the severe limitations of human clinical data for alopecia areata. The human evidence consists of only two small, uncontrolled reports: one retrospective cohort study (n ≈ 39 AA patients) and one single-patient case report. This represents very low-certainty evidence by GRADE criteria and is insufficient to support any strong conclusions about either efficacy or safety in human alopecia areata.

The small sample size (40 patients total) creates two critical problems. First, for safety assessment, 40 patients provide grossly inadequate statistical power to detect uncommon adverse events. While no serious adverse events were reported, the sample size is insufficient to exclude harms occurring at frequencies ≤ 2%—a threshold below which many clinically important adverse events occur. For context, anaphylaxis to biologic agents typically occurs at 0.1–1% frequency and opportunistic infections with immunosuppressants at 1–5% frequency. The statement “no serious adverse events were observed” should not be misinterpreted as evidence of safety; rather, it reflects inadequate sample size to detect rare but potentially important events.

Second, for efficacy assessment, the absence of control groups prevents attribution of observed improvements to exosome therapy. Alopecia areata exhibits high spontaneous remission rates, particularly in patchy forms, where 34–50% of patients achieve spontaneous recovery within one year without any treatment. Without concurrent controls, reported hair regrowth could represent natural disease fluctuation rather than treatment effect. Additionally, retrospective designs introduce substantial selection bias (patients with favorable outcomes more likely to be captured and reported), detection bias (unblinded assessment of subjective outcomes like hair density), and confounding (patients receiving exosomes may differ systematically in disease severity, duration, or other prognostic factors from the broader AA population).

Therefore, the human clinical data—while hypothesis-generating and supporting the feasibility of exosome administration—cannot support definitive conclusions about efficacy or safety. The evidence hierarchy for this review is as follows: (1) robust preclinical animal data providing proof of concept with controlled experiments, reproducible effects, and clear mechanistic basis; (2) very limited, very low-certainty human case reports that are hypothesis-generating only; (3) an urgent need for rigorous clinical trials with adequate sample sizes, control groups, and systematic outcome assessment. Readers and clinicians should not overinterpret preliminary human findings or consider them sufficient for clinical decision-making.

This systematic review synthesizes current evidence on exosome-based therapies for alopecia areata, revealing a promising but nascent field characterized by strong biological plausibility, encouraging preclinical results, and limited but positive early human experience. The key findings can be summarized as follows:

- Good mechanistic foundation: EVs or sEVs from multiple sources (MSCs, dermal papilla cells, platelets) consistently activate hair regenerative pathways (Wnt/β-catenin, PI3K/Akt) and exert immunomodulatory effects relevant to AA pathogenesis (reduction of IFN-γ signaling, modulation of CD8+ T cells, promotion of immune privilege) [1,4,5].
- Robust preclinical efficacy: controlled animal studies demonstrate substantial hair regrowth (50–99% coverage improvement), restoration of follicular architecture, and dampening of perifollicular inflammation [17,18,19] in validated AA animal models, with particularly potentially good results when exosomes are used as delivery vehicles for JAK inhibitors. Although the combination of exosome-based therapies with JAK inhibitors is conceptually attractive—potentially uniting immunosuppressive and regenerative mechanisms—there are currently no human studies in alopecia areata evaluating this approach, and its clinical utility remains speculative.
- Preliminary human efficacy signals: small clinical reports show meaningful improvements in hair density (9–31 hairs/cm^2^), SALT score reductions, and patient satisfaction, though these findings require validation in controlled trials.
- Significant heterogeneity and methodological limitations: variability in exosome source, isolation methods, characterization, dosing, and delivery routes precludes meta-analysis and limits comparative effectiveness assessment. The absence of randomized controlled trials represents a critical evidence gap.

### 4.2. Interpretation in Context of Current AA Therapeutics

The therapeutic landscape for alopecia areata has evolved significantly with the recent FDA approvals of JAK inhibitors [12,13] (baricitinib [12] in 2022, ritlecitinib [13] in 2023), which target the interferon-driven pathology at its core. These oral agents achieve SALT score improvements of 50% [12,57] or greater in 30–40% of patients with severe AA, representing a major advance over traditional therapies. However, challenges remain: not all patients respond adequately, relapse is common upon discontinuation, long-term safety monitoring is required, and cost and access barriers limit widespread adoption.

Exosome therapy offers several potential advantages as a complementary or alternative approach. First, exosomes provide localized immunomodulation and regenerative signaling directly to affected follicles, potentially minimizing systemic exposure and side effects—a critical consideration for pediatric patients, those with comorbidities, or patients requiring long-term management. Second, the regenerative mechanisms (Wnt/β-catenin activation, stem cell stimulation, angiogenesis promotion) are complementary rather than redundant to JAK inhibition [12,13], suggesting potential for combination strategies. The preclinical data showing enhanced efficacy of baricitinib [12] when delivered via exosomes supports this concept. Third, exosome therapy may be particularly suited for early-stage or mild-to-moderate AA, where the risk–benefit calculus for systemic immunosuppression is less favorable.

However, several critical unknowns must be addressed before exosome therapy can be positioned within the AA treatment algorithm. The optimal exosome source remains unclear: while ADSC and UC-MSC EVs [21,51] dominate current research due to availability and manufacturing scalability, dermal-papilla-derived exosomes may offer superior hair inductive properties due to their native tissue-specific cargo. Dose–response relationships are poorly characterized; existing studies use widely varying concentrations with no systematic comparison. The durability of response is unknown beyond 6 months. Finally, the comparative efficacy versus established treatments (intralesional corticosteroids [10,11,56], contact immunotherapy, oral JAK inhibitors) has not been evaluated.

The safety assessment limitations and future needs are as follows: despite the encouraging short-term safety profile, important limitations must be acknowledged. The total patient exposure is small (n = 40), limiting the ability to detect rare adverse events that might occur at frequencies of <2%. Follow-up durations are insufficient to assess long-term safety concerns, including potential oncogenic risks, given that exosomes carry bioactive molecules capable of influencing cell proliferation and differentiation pathways [14,15]. No studies reported systematic screening for anti-exosome antibodies or immune sensitization, which could theoretically develop with repeated administrations, particularly with allogenic or xenogeneic sources (bovine colostrum). The use of xenogeneic material like bovine colostrum EVs presents unique regulatory hurdles and heightened theoretical risks of immune rejection or sensitization that mandate stringent safety evaluation and specific immunogenicity testing before clinical translation. The variability in exosome preparation methods across studies, ranging from ultra-centrifugation to commercial precipitation kits, raises questions about consistency in purity and the potential for contaminant-related adverse effects [58]. Future studies should include the following: (1) larger cohorts with statistical power to detect adverse events occurring at 1–5% frequency; (2) extended follow-up (≥24 months) to assess durability of safety; (3) systematic collection of patient-reported outcome measures specifically addressing quality of life and treatment burden; (4) standardized adverse event reporting aligned with Common Terminology Criteria for Adverse Events (CTCAE); (5) investigation of immunogenicity through serial antibody testing; and (6) post-marketing surveillance registries if exosome therapies receive regulatory approval. Particular attention should be paid to vulnerable populations, including pregnant women, immuno-compromised patients, and children, for whom safety data are currently absent.

In terms of angiogenesis and follicular microenvironment support, exosome-mediated increases in VEGF [17,51], HGF, and IGF-1 enhance perifollicular vascularization and provide trophic support for hair follicle stem cells and matrix keratinocytes. This angiogenic effect may be particularly important for reversing the capillary rarefaction [17] observed in chronic AA.

Regarding stem cell activation and proliferation, the ability of exosomes to enhance hair follicle stem cell proliferation [16,32] while reducing apoptosis (via the novel-238a-CASP9 axis [32] and other mechanisms) provides a dual benefit: expanding the stem cell pool available for follicular regeneration while protecting these cells from immune mediated destruction.

These mechanistic insights suggest that EV or sEV therapy may be most effective when administered early in the disease course, before irreversible follicular damage occurs, and in combination with agents that address the primary autoimmune driver (such as JAK inhibitors for initial immune suppression followed by exosomes for sustained regeneration and immune privilege [1,4,5] maintenance).

### 4.3. Methodological Considerations and Standardization Needs

The marked heterogeneity identified in this review highlights the urgent need for standardization across the exosome therapy pipeline.

In terms of exosome characterization, future studies must adhere to MISEV (Minimal Information for Studies of Extracellular Vesicles) 2018/2023 guidelines, reporting (1) particle size distribution and concentration (measured by NTA or similar); (2) protein quantification; (3) positive markers (CD9, CD63, CD81, TSG101/ALIX [14]); (4) negative markers (GM130, calnexin); (5) source cell verification; and (6) cargo analysis (miRNA profiling, proteomic characterization) when mechanistic claims are made. Without this information, it is impossible to compare studies or determine which exosome populations are responsible for observed effects.

In terms of dose standardization, the field needs consensus on dose units and a systematic dose-ranging investigation. Reporting both particle number (e.g., 1 × 1010 particles) and protein content (e.g., 50 µg) provides complementary information about product composition. Dose–response studies should evaluate clinically relevant outcomes across at least 3–4 dose levels to identify optimal therapeutic windows.

In terms of delivery optimization, the reviewed literature suggests multiple viable delivery routes like intradermal injection, microneedling-assisted application, and topical formulations, but comparative data are lacking. Intradermal injection provides precise dose control and proven follicular targeting but is invasive and requires trained practitioners. Microneedling may enhance penetration while being more patient-friendly. Emerging technologies, such as dissolving microneedle patches [47] and hydrogel-based sustained-release formulations, warrant investigation for their potential to improve patient compliance and therapeutic durability.

In terms of outcome assessment, future AA trials should include a standardized core outcome set: SALT score (primary efficacy endpoint for most trials), trichoscopic parameters (hair density [hairs/cm^2^], shaft diameter, yellow dots), global photography with blinded expert assessment, patient-reported outcomes (validated instruments such as the Alopecia Areata Quality of Life index) [4], and biomarker assessments (perifollicular immune infiltrate, anagen/telogen ratio). Follow-up should extend to at least 12 months to assess durability and relapse rates.

### 4.4. Translational Imperative and Call for Clinical Trials

While the current human data are limited and largely anecdotal, the convergence of compelling preclinical efficacy, mechanistic plausibility, and favorable safety profiles presented in this review collectively establishes a robust scientific foundation for clinical translation. The consistency of regenerative and immunomodulatory effects across diverse exosome sources and delivery strategies underscores the therapeutic potential of exosome-based interventions in alopecia areata. This synthesis supports an urgent need for rigorously designed phase II randomized controlled trials (RCTs) to validate clinical efficacy, define optimal dosing regimens, and ensure long-term safety. Future trials should incorporate stratification by disease severity and chronicity, adhere to standardized exosome characterization per MISEV guidelines, and utilize validated outcome measures such as SALT scores, trichoscopy, and patient-reported outcomes. Without such trials, the field risks stagnation at the preclinical proof-of-concept stage despite promising early signals.

### 4.5. Limitations of the Study

Several limitations should be considered when interpreting these findings. First, the paucity of high-quality clinical data limits the strength of conclusions regarding human efficacy. The two included clinical reports are uncontrolled, involve small samples, and use non-standardized interventions and outcomes. Publication bias likely favors positive results. Second, we included mechanistic studies using non-AA hair models to provide biological context, but the relevance of findings from androgenetic alopecia or normal follicle studies to the immune-mediated pathophysiology of AA is uncertain. Third, language restriction (English only) and date limitation (2020–2025) may have excluded relevant evidence, though a hand-search of references suggests that earlier foundational work was adequately captured through cited reviews. Fourth, the lack of standardized reporting precluded meta-analysis; our synthesis is qualitative, and effect size estimates should be interpreted cautiously. A notable limitation in the reviewed studies is the sparse reporting on participant demographics. Variables such as age, sex, ethnicity, and disease chronicity were often omitted, restricting the generalizability of findings. Given the immunological and psychosocial nuances of AA across populations, future trials must stratify outcomes by these parameters. Finally, long-term safety data are absent, and rare adverse events would not be detected in studies of this size. Additionally, while we did not perform a meta-analysis, a more quantitative synthesis of preclinical animal data may be feasible. Several studies reported comparable endpoints, such as percentage of hair coverage and follicle counts, which could support future meta-analytic efforts. However, lack of variance reporting, inconsistent sample sizes, and methodological diversity across animal models constrained the validity of such pooling in this iteration. A major limitation affecting comparability and translational relevance is the insufficient characterization of exosome preparations across studies. Most investigations did not fully adhere to MISEV criteria, with frequent omissions in particle quantification, EV markers, purity controls, and dose standardization. This lack of reporting limits cross-study comparability and constrains conclusions about optimal sources, doses, and delivery methods. Future studies should incorporate full MISEV-based profiling to enable reproducible clinical translation. General hair regeneration studies (e.g., AGA or depilation models) emphasize regenerative pathways, while AA models involve immune-mediated follicular destruction. A critical limitation is marked heterogeneity in exosome dose reporting across preclinical studies. Studies employed different quantification metrics without conversion factors, precluding direct efficacy comparison. The inability to establish dose–response relationships is particularly problematic—we cannot determine whether higher doses produce greater efficacy, whether there is a plateau effect, or identify an optimal dose range. This complicates clinical trial design: should human trials use protein-based dosing (most common preclinically), particle-based (more biologically relevant), or another metric? Without standardized dose reporting, rational clinical dose selection is impaired.

### 4.6. Future Research Directions

To translate the promising preclinical evidence into clinical practice, the following research priorities are recommended: rigorous phase II randomized controlled trials; adequately powered (≥60 patients), double-blind, placebo-controlled trials with standardized exosome products, clearly defined dosing regimens, and validated outcome measures. Stratification by baseline severity (mild/moderate vs. severe) and disease duration (<1 year vs. >1 year) will enable subgroup analyses. Primary endpoints should include SALT score change at 24 weeks, with secondary endpoints of hair density, patient reported outcomes, and safety.

### 4.7. Comparative Effectiveness Research

Head-to-head trials comparing exosome therapy to established treatments (intralesional corticosteroids [10,11,59] for mild-moderate AA; JAK inhibitors for severe AA) are essential to define the clinical niche. Non-inferiority trial designs may be appropriate for positioning exosomes as a safer alternative for specific populations (e.g., children, patients with comorbidities).

In terms of combination therapy studies, experimental evidence supports synergy between JAK inhibition [9,12,13,57,59,60,61] and exosome therapy. Trials examining sequential treatment (JAK inhibitor induction followed by exosome maintenance) or simultaneous combination therapy could reveal optimal treatment paradigms. In terms of biomarker-driven patient selection, not all AA patients may respond equally to exosome therapy. Investigating baseline predictors of response (e.g., perifollicular immune infiltrate density, specific cytokine profiles, baseline Wnt pathway activity) could enable precision medicine approaches [58,59,60,61].

Product optimization and manufacturing standardization—comparative studies of different exosome sources (ADSC vs. UC-MSC vs. dermal-papilla-derived), isolation methods, and storage conditions—are needed. Development of good manufacturing practice (GMP), compliant production protocols, and stability testing will be prerequisites for regulatory approval [62,63].

### 4.8. Long-Term Follow-Up Studies

Durability of response and maintenance therapy requirements must be characterized. Registries tracking patients for 2–5 years post-treatment will provide essential real-world evidence about relapse rates, the need for repeat treatments, and late-emerging adverse events. Economic evaluation—cost-effectiveness analyses comparing exosome therapy to current treatments, considering both direct medical costs and quality-of-life benefits—will inform healthcare policy and reimbursement decisions.

## 5. Conclusions

This systematic review suggests that exosome-based therapy may offer a promising and biologically plausible approach for the treatment of alopecia areata. Preclinical studies consistently demonstrate beneficial effects in both in vitro and animal models, and early clinical reports provide preliminary signals of efficacy. While the mechanistic data are encouraging, highlighting pathways potentially relevant to AA pathogenesis, the majority of evidence to date comes from non-randomized and preclinical studies. As such, further research, particularly well-controlled clinical trials, is needed to establish the therapeutic potential and translational relevance of exosome-based interventions in AA.

For clinicians managing AA, exosome therapy currently represents an experimental modality that should be considered only within approved research protocols or in jurisdictions where regulatory pathways permit innovative therapies under appropriate oversight. The favorable safety profile observed to date is reassuring, but long-term data are lacking. Patients inquiring about exosome therapy should be counseled about the preliminary nature of the evidence, the absence of FDA-approved exosome products for AA, and the substantial heterogeneity in available formulations. Clinicians should emphasize that established therapies (topical immunotherapy, intralesional corticosteroids, JAK inhibitors) remain first-line options supported by higher-quality evidence. The regulatory landscape for exosome therapy remains underdeveloped. It is unclear whether exosome preparations will be classified as biologics, drugs, or advanced therapy medicinal products (ATMPs). This ambiguity complicates trial design and commercialization. Furthermore, ethical considerations, particularly concerning allogeneic or xenogeneic sources such as bovine colostrum, must be addressed. These include potential immunogenicity, informed consent for donor tissues, and safety assurances regarding cross-species transmission. The field has reached an inflection point where further observational studies and mechanistic investigations, while valuable, will provide diminishing returns without rigorous clinical trials. The priority must shift to well-designed randomized controlled trials with standardized interventions, validated endpoints, and adequate follow-up. Regulatory agencies and funding bodies should prioritize support for such trials, recognizing the potential public health impact given AA’s prevalence (affecting 2% of the population) and substantial quality-of-life burden. For exosome therapy to move from bench to bedside, several critical steps must be accomplished: (1) establishment of GMP-compliant [58] manufacturing standards and quality control criteria; (2) completion of phase II dose-finding and safety trials in well-defined AA populations; (3) demonstration of efficacy in adequately powered phase III trials using standardized, clinically meaningful endpoints; and (4) post-marketing surveillance to detect rare adverse events and characterize real-world effectiveness.

## Figures and Tables

**Table 1:** Studies included in the systematic review.

Study	Study Type	Exosome Source	Study Population/Model	Intervention Protocol
Zöller et al., 2018 [20]	Experimental (animal)	Bone marrow cells	Female C3H/HeJ mice, alopecia areata (AA) model	Intravenous injection, 100 µg, twice/week, 10 weeks
Bento et al., 2025 [21]	Clinical (case report)	Rose stem cell	39-year-old male, unifocal AA	6 monthly sessions, 20 mg/vial, laser-assisted
Tang et al., 2024 [18]	Experimental (animal)	Mesenchymal stem cell (MSC) exosomes	AA mouse model (interferon-gamma-induced)	Local injection, baricitinib-loaded extracellular vesicles (EVs)
Li et al., 2022 [22]	Mixed (in vitro, animal)	Adipose-derived stem cells	Dermal papilla cells (DPCs) (in vitro), C57BL/6 mice	Subcutaneous injection, alone/with minoxidil
Cho B.S., 2023 [23]	Mixed (in vitro, in vivo, clinical)	Adipose stem cell	29 subjects (AA, androgenetic alopecia), DPCs	Microneedling + topical adipose stem cell extract (ASCE), 10 sessions/6 months
Hu et al., 2024 [24]	Experimental (animal)	Umbilical cord MSC	AA mice	No mention found
Cestari et al., 2025 [25]	Mixed (review)	MSC (primed/engineered)	Preclinical/clinical studies	Topical/intradermal, no mention found
Poddar et al., 2025 [26]	Mixed (systematic review)	Various	In vitro, preclinical, clinical	No mention found
Paiewonsky et al., 2022 [27]	Mixed (review)	No mention found	In vivo/in vitro preclinical	No mention found
Nunez et al., 2025 [28]	Clinical (review)	No mention found	12 clinical studies	No mention found
Park et al., 2022 [29]	Clinical (retrospective)	Adipose-derived stem cell	39 patients, alopecia (type not specified)	Intradermal injection, 12 weeks
Kost et al., 2022 [30]	Mixed (review)	Multiple	Preclinical, pilot clinical	No mention found
Dairov et al., 2025 [31]	Review (clinical)	MSC	Clinical studies, case reports	No mention found
Zhao et al., 2025 [32]	In vitro	Dermal papilla cell	Hair follicle stem cells (HFSCs) (cell culture)	DPC-Exos + CS-COL17A1, dose-dependent
Cheng et al., 2024 [33]	Mixed (review)	DPC, MSC	Preclinical, in vitro	No mention found
Hu et al., 2020 [34]	In vitro	Dermal papilla spheroids	Dermal papilla (DP) cells (spheroid culture)	No mention found
Chen et al., 2025 [35]	In vitro	Umbilical cord MSC	Human hair dermal papilla cells (HHDPCs) (cell culture)	No mention found
Rajendran et al., 2022 [36]	In vitro/ex vivo	Bone marrow MSC	DP, outer root sheath (ORS) cells, human hair follicles (HFs)	2–10 µg/mL, 24 h, ex vivo
Schaffer et al., 2025 [37]	Mixed (experimental, clinical)	MSC, adipose, others	Animal models, human	Microneedle patch, topical, intradermal
Wu and Tang, 2025 [38]	Review	No mention found	Literature review	No mention found
Li et al., 2022 [39]	Review	MSC	Narrative review	No mention found
Zhou et al., 2018 [16]	Mixed (animal, in vitro)	Dermal papilla cell	Mice, outer root sheath cells (ORSCs)	Intradermal injection
Salhab et al., 2022 [40]	Mixed (review)	Adipose-derived stem cell	Mice, human DPCs (hDPCs)	Subcutaneous, not detailed
Guermazi et al., 2024 [41]	Review	No mention found	Review	No mention found
Anudeep et al., 2022 [42]	Mixed (review)	Multiple	Clinical, preclinical, in vitro	No mention found
Palkina et al., 2024 [43]	Review	No mention found	Review	No mention found
Ku et al., 2023 [44]	Review	Bone marrow, placental, adipose, umbilical	Review	No mention found
Nahm et al., 2025 [45]	Review (clinical)	Human stem cell	Review	No mention found
Chen et al., 2023 [46]	Experimental (animal)	No mention found	11 animal models	No mention found
Frasier et al., 2024 [47]	Review	Stem cell, keratinocyte, fibroblast	Review	No mention found
Sun et al., 2025 [48]	Review	No mention found	Review	No mention found
Gupta et al., 2023 [49]	Mixed (experimental, clinical)	No mention found	Preclinical, experimental clinical	No mention found
Queen and Avram, 2025 [50]	Review	No mention found	Review	No mention found
Kim et al., 2022 [19]	Preclinical (animal)	Bovine colostrum	C57BL/6 mice	Intradermal, 100 µg, every other day, 19 days
Nilforoushzadeh et al., 2021 [51]	In vitro	Adipose stem cell, platelet-rich plasma (PRP)	hDPCs, ORSCs	25–100 µg/mL, no mention found
Nilforoushzadeh et al., 2020 [52]	In vitro	Human hair outer root sheath cells (HHORSCs), platelet lysis	hDPCs, HHORSCs	2, 50, 100 µg/mL; no mention found
Zhang et al., 2025 [53]	Mixed (clinical, experimental)	Adipose-derived stem cell	Androgenetic alopecia, AA patients	Microneedling, 12 weeks
Matwiejuk et al., 2025 [54]	Mixed (experimental, clinical)	Bone marrow MSC (BM-MSC), human umbilical cord blood MSC (hUCB-MSC)	Animal models, small clinical	Topical, intraperitoneal, daily 10 days
Mao et al., 2024 [55]	Mixed (animal, in vitro)	Umbilical cord MSC	C57BL/6 mice, fibroblasts	200 µg/mL, no mention found
Cho, 2023 [56]	Mixed (in vitro, in vivo, clinical)	Adipose stem cell	29 subjects (AA, androgenetic alopecia), DPCs	Microneedling + topical ASCE, 10 sessions/6 months

AA—specific experimental evidence.

**Table 2:** Summary of preclinical animal studies in alopecia areata models.

Study	Model	Exosome Source	Intervention	Hair Regrowth	Mechanism
Tang et al., 2024 [18]	IFN-γ-induced AA (C3H/HeJ)	Baricitinib-loaded EVs	Subcutaneous injection	62 ± 11% coverage	JAK-STAT inhibition
Kim et al., 2022 [19]	Telogen-arrest model	Colostrum-derived	Topical application	Anagen acceleration	Wnt/β-catenin activation
Zöller et al., 2018 [20]	C3H/HeJ AA model	Bone marrow MSC	Intravenous (100 µg)	30% (totalis), 16% (partialis), 95–100% (incipient)	Treg expansion, immunomodulation
Hu et al., 2024 [24]	AA mice	UC-MSC	No details reported	Improved (not quantified)	Keratinocyte proliferation
Li et al., 2022 [22]	C57BL/6 mice	ADSC	Subcutaneous ± minoxidil	Improved growth, increased follicles	Wnt/β-catenin, miR-22

AA—alopecia areata, UC—umbilical cord, MSC—mesenchymal stromal cell, ADSC—adipose-derived stem cell.

**Table 3:** SYRCLE risk of bias in animal studies.

Study	Sequence Generation	Baseline Characteristics	Allocation Concealment	Random Housing	Blinding of Investigators	Random Outcome Assessment	Blinding of Outcome Assessor	Incomplete Outcome Data	Selective Outcome Reporting	Other Bias	Overall Risk
Tang et al., 2024 [18]	Unclear	Low	Unclear	Unclear	High	Unclear	High	Low	Unclear	High	High
Kim et al., 2022 [19]	Low	Low	Unclear	Low	Low	Low	Low	Low	Low	Low	Low

**Table 4:** Characteristics of included clinical studies of alopecia areata.

Study	Design	Sample Size	Exosome Source	Delivery Method	Primary Outcome	Follow-Up
Park et al., 2022 [29]	Retrospective cohort	n = 39 (AA subset)	ADSC	Intradermal injection	Hair density (hairs/cm^2^)	6 m
Bento et al., 2025 [21]	Case report	n = 1	Plant-derived	Microneedling + topical	Photographic assessment	6 m

Notes: n—number, ADSC—adipose-derived stem cell, m—month.

**Table 5:** ROBINS-I tool for risk-of-bias assessment.

Study	Design	Bias Due to Confounding	Bias in Selection of Participant	Bias in Classification of Intervention	Bias Due to Deviations from Intended Intervention	Bias Due to Missing Data	Bias in Measurement of Outcomes	Bias in Selection of Reported Results	Overall Risk of Bias
Park et al., 2022 [29]	Retrospective cohort	Moderate	Low	Low	Moderate	Low	Moderate	Low	Moderate
Bento et al., 2025 [21]	Case report	Serious	Low	Moderate	Serious	Low	Moderate	Serious	Serious

**Table 6:** Key mechanistic pathways activated by exosome therapy.

Study	Mechanisms of Action
Zöller et al., 2018 [20]	Regulatory T cell (Treg) expansion, reduced T helper proliferation, increased FoxP3/arginase 1 messenger RNA (mRNA)
Bento et al., 2025 [21]	No mention found
Tang et al., 2024 [18]	Janus kinase-signal transducer and activator of transcription (JAK-STAT) inhibition, Wnt/β-catenin upregulation
Li et al., 2022 [22]	Wnt/β-catenin, microRNA-22 (miR-22), tumor necrosis factor-alpha (TNF-α) downregulation
Cho B.S., 2023 [23]	Antiaging, anti-inflammatory, dermal papilla cell effects
Hu et al., 2023 [24]	Keratinocyte proliferation/migration
Cestari et al., 2025 [25]	No mention found
Poddar et al., 2025 [26]	Dermal papilla cell stimulation, angiogenesis, inflammation modulation
Paiewonsky et al., 2022 [27]	No mention found
Nunez et al., 2025 [28]	No mention found
Park et al., 2022 [29]	No mention found
Kost et al., 2022 [30]	Wnt/β-catenin, TNF-α, vascular endothelial growth factor (VEGF), FoxP3/arginase 1
Dairov et al., 2025 [31]	No mention found
Zhao et al., 2025 [32]	hsa-novel-238a-CASP9 axis, hair follicle stem cell migration/viability
Cheng et al., 2024 [33]	No mention found
Hu et al., 2020 [34]	Wnt/β-catenin, miR-218-5p
Chen et al., 2025 [35]	Phosphoinositide 3-kinase/protein kinase B (PI3K/Akt), β-catenin, cyclin D1
Rajendran et al., 2022 [36]	Wnt/β-catenin, dermal papilla/outer root sheath proliferation, keratin expression
Schaffer et al., 2025 [37]	Wnt/β-catenin, Sonic Hedgehog, VEGF, insulin-like growth factor-1 (IGF-1), M1→M2 macrophage
Wu and Tang, 2025 [38]	No mention found
Li et al., 2022 [39]	No mention found
Zhou et al., 2018 [16]	Wnt (β-catenin), Sonic Hedgehog (Shh), outer root sheath cell proliferation/migration
Salhab et al., 2022 [40]	Wnt/β-catenin, transforming growth factor-beta (TGF-β), extracellular signal-regulated kinase (Erk), Akt, VEGF, miR-22
Guermazi et al., 2024 [41]	Dermal papilla cell proliferation, inflammation modulation
Anudeep et al., 2022 [42]	Wnt/β-catenin, bone morphogenetic protein (BMP), Hedgehog, Notch, VEGF, platelet-derived growth factor (PDGF), IGF-2
Cho, 2023 [56]	Antiaging, anti-inflammatory, dermal papilla cell effects

**Table 7:** Safety profile of exosome therapy across clinical and preclinical studies.

Adverse Event Type	Frequency	Severity	Resolution
Injection-site discomfort	68% (intradermal)	Mild	24–48 h
Transient erythema	41%	Mild	2–5 days
Pruritus	2.3% (1 patient)	Mild	3 days
Serious adverse events	0%	None reported	N/A

**Table 8:** MISEV compliance summary for included AA studies.

Study	Particle Size	Particle Count (NTA)	Protein Content	Positive Markers	Negative Markers	Source Cell Verified
Park et al., 2022 [29]	No	No	Yes	Unclear	No	Yes
Bento et al., 2025 [21]	No	No	Yes	No	No	Yes
Tang et al., 2024 [18]	Yes	Yes	Yes	Yes	No	Yes
Kim et al., 2022 [19]	Yes	Yes	Yes	Yes	No	Yes
Zöller et al., 2018 [20]	No	No	Yes	Unclear	No	Yes
Li et al., 2022 [22]	Yes	No	Yes	Unclear	No	Yes
Hu et al., 2024 [24]	No	No	Yes	Unclear	No	Yes

## Data Availability

No new data were created or analyzed in this study. Data sharing is not applicable to this article.

## Abbreviations

The following abbreviations are used in this manuscript:

AA	Alopecia areata
ADSC	Adipose-derived stem cell
AKT	Protein Kinase B
ALP	Alkaline phosphatase
APC	Antigen-presenting cell (or article processing charge in a funding context)
ARRIVE	Animal Research: Reporting of In Vivo Experiments
AST	Aspartate aminotransferase
ALT	Alanine aminotransferase
BM-MSC	Bone marrow mesenchymal stem cell
BUN	Blood urea nitrogen
CASP9	Caspase 9
CD	Cluster of differentiation (e.g., CD8, CD9, CD63, CD81)
CRP	C-Reactive Protein
CTCAE	Common Terminology Criteria for Adverse Events
DPCP	Diphenylcyclopropenone
DKK1	Dickkopf-related protein 1
DP	Dermal papilla
ESR	Erythrocyte sedimentation rate
EV	Extracellular vesicle
FDA	Food and Drug Administration (U.S.)
GRADE	Grading of Recommendations Assessment, Development and Evaluation
GMP	Good manufacturing practice
HGF	Hepatocyte Growth Factor
ICTRP	International Clinical Trials Registry Platform
IFN-γ	Interferon-gamma
IGF-1	Insulin-like Growth Factor 1
IL	Interleukin (e.g., IL-10, IL-15)
ITT	Intention to treat
JAK	Janus Kinase
JAK-STAT	Janus kinase-signal transducer and activator of transcription
LEF1	Lymphoid Enhancer-binding Factor 1
MEDLINE	Medical Literature Analysis and Retrieval System Online
MISEV	Minimal Information for Studies of Extracellular Vesicles
miRNA	microRNA
mRNA	Messenger RNA
MSC	Mesenchymal stromal/stem cell
NICE	National Institute for Health and Care Excellence
NK	Natural killer (cells)
NKG2D	Natural killer group 2D
NTA	Nanoparticle tracking analysis
PCNA	Proliferating cell nuclear antigen
PI3K	Phosphoinositide 3-kinase
PICOS	Population, Intervention, Comparator, Outcomes, Study design
PRISMA	Preferred Reporting Items for Systematic Reviews and Meta-Analyses
PRP	Platelet-rich plasma
RCT	Randomized controlled trial
RoB	Risk of bias
ROBINS-I	Risk Of Bias In Non-randomized Studies of Interventions
SADBE	Squaric acid dibutylester
SALT	Severity of Alopecia Tool
sEV	Small extracellular vesicle
SFRP2	Secreted Frizzled-related Protein 2
SHH	Sonic hedgehog
STAT	Signal Transducer and Activator of Transcription
SYRCLE	Systematic Review Centre for Laboratory Animal Experimentation
TGF-β	Transforming Growth Factor-beta
Th	T helper (cells) (e.g., Th1, Th2, Th17)
TSG101	Tumor Susceptibility Gene 101
UC-MSC	Umbilical cord mesenchymal stem cell
VEGF	Vascular Endothelial Growth Factor
WHO	World Health Organization
Wnt	Wingless-related integration site (signaling pathway)
